# Magnetic Induction Spectroscopy for Permeability Imaging

**DOI:** 10.1038/s41598-018-25507-4

**Published:** 2018-05-04

**Authors:** Lu Ma, Manuchehr Soleimani

**Affiliations:** Engineering Tomography Laboratory, Department of Electronic and Electrical Engineering, University of Bath, Claverton Down, BA2 7AY UK

## Abstract

The structural health monitoring of ferromagnetic materials is essential for controlling product quality. This paper presents the first known characterisation, utilising magnetic induction spectroscopy, of the magnetic permeability of ferromagnetic materials, a property that can facilitate such monitoring. Magnetic induction coils are interrogated by a sweep of excitation frequencies in the range of 1–100 kHz, with amplitudes of induced voltages collected from both non-magnetic and ferromagnetic specimens, and their associated spectral permeability imaged. The reconstructed images show that magnetic permeability can be reconstructed using frequency difference imaging in the investigated frequency range. As such, the relative magnetic permeability of a ferromagnetic specimen can be reconstructed without needing a time difference measurement. This provides a robust imaging approach for the material characterisation of ferromagnetic specimens.

## Introduction

Ferromagnetic materials such as steel are an integral component of modern infrastructure. Monitoring the state of these materials and their structural integrity is an essential, but challenging, task. This study demonstrates that the characterisation of ferromagnetic materials can be achieved via their frequency-dependent magnetic permeability, if using a non-invasive and non-contact magnetic induction spectroscopy imaging system.

For a given imaging specimen, perturbations in electromagnetic fields caused by a change of electromagnetic properties can be measured as the voltages induced in magnetic induction coils. An image can be reconstructed from these voltages by solving the inverse problem. For imaging conductivity, a time difference technique is commonly used^1^. Changes in the induced voltages are the result of changes in conductivity arising from material structure^2^, the external environment (in either the spatial or time domains)^3^ or due to frequency-dependent pathophysiological information^4^. The permittivity can also be derived from, and has an effect on, both the real and imaginary part of the induced voltages^5^. Magnetostatic permeability reconstruction using imaging techniques has only been shown in the time difference domain^6^. Frequency-dependent magnetic permeability has been observed using other non-imaging techniques, such as impedance measurements picked up by coils placed closely to the ferromagnetic specimen^7^, a four point alternating current potential drop measurement technique^8^, and a direct measurement of the hysteresis curve of the material of interest by *H-*coils^9^.

This paper presents the evidential basis of frequency-dependent permeability from magnetic induction measurements and uses a frequency difference technique to reconstruct the images of the amplitude of complex magnetic permeability. Within the frequency range of 1–100 kHz, and in the presence of both non-magnetic and ferromagnetic specimens, it is shown that only ferromagnetic specimens have a frequency difference response with regard to their reconstructed image. This is, to the best of our knowledge, the first report of this nature employing both magnetic induction measurements and a tomographic approach.

## Measurement System

An eight-channel magnetic induction tomography (MIT) system is used for frequency spectroscopy imaging. The system comprises a signal generator, a front-end electronics component, multiplexers, a data acquisition unit and an array of inductive coils. The inductive sensor is a single layer 50-turn air-cored inductive coil with a radius of 0.021 m and a side length of 0.014 m, as previously described^10^. The excitation signal is provided by a Direct Digital Synthesizer (DDS) AD9834 operating from 1 to 100 kHz. The front-end electronics component contains eight VO14642 multiplexers as solid state relays that drive the transmission coils and the receiving coils sequentially. The maximum excitation current is 100 mA. The induced voltage is amplified by a TLE2071 amplifier. At a maximum frequency of 100 kHz, the maximum voltage measured from the neighbouring coils is approximately 5 V after the amplification. The induced voltage is sampled by an on-board 10-bit ADC. The amplitude of the induced voltage is collected from Arduino and communicated to a host PC via a USB connection for image reconstruction. The system has a frame rate of 0.2 frame/s, where one frame includes driving the excitation coils one at a time and measuring the receiving coils in sequence, transferring the measurements to a host PC, reconstructing and displaying an image using measurement data. Given that the frequency sweep interval is, at minimum, 5 s, it is safe to assume that any material response due to frequency differences is settled before the next interrogation within the ferromagnetic specimen under investigation.

## Methods

MIT utilises an array of inductive coils, distributed equally around an imaging region, to visualise the electromagnetic property distribution of an imaging specimen. The governing equation for the MIT forward model^11^ is given as equation 1, where *μ* is the permeability, *A* is the magnetic vector potential, *ω* is the angular frequency, *σ* is the electrical conductivity, and *J*_*S*_ is the excitation current density. Equation 1 is solved using the edge finite element method; the representation of the source current density using an electric vector potential ensured the divergence free of electric current.

1 $$\nabla \times \frac{1}{\mu }\nabla \times A+i\omega \sigma A={J}_{s}$$

The imaging principle is based on the laws of induction and eddy currents, which are induced in an AC magnetic field. The induced voltages in receiving coils are calculated using volume integration over the receiving coil region^12^ as equation 2, where *J*_0_ is a unitary current density following the strands of the receiving coil.

2 $${V}_{R}=-\,i\omega {\int }_{V}A\cdot {J}_{0}dV$$

The imaging process requires formulating the sensitivity distribution. This describes the relationship between the changes in complex electric and magnetic fields in a discretized perturbation region Ω and the changes in electromagnetic properties^13^. These changes can be measured from the voltages induced due to a given excitation coil *m* and a receiving coil *n*. For a given system, the excitation source *I*_*m*_, *I*_*n*_ and the angular frequency *ω* are known. The excitation source current *I*_*m*_ is the surface integral of *J*_*S*_ in a cross section of the excitation coil wire.

3 $$\frac{\partial {V}_{mn}}{\partial \mu }=-\frac{i\omega }{{I}_{m}{I}_{n}}{\int }_{{\rm{\Omega }}}{H}_{m}\cdot {H}_{n}d{\rm{\Omega }}$$

In time difference MIT imaging, the electromagnetic properties and excitation frequency are assumed to be constant at *t*_1_ and *t*_2_. It is common practice to use the induced voltages in a free space as reference, where *μ*_0_, *σ*_0_ and *ε*_0_ refer to the permeability, conductivity and permittivity in a free space, respectively.

4 $${\rm{\Delta }}{V}_{t1,t2}(\delta \mu ,\delta \sigma ,\delta \varepsilon )={V}_{t2}(\mu ,\sigma ,\varepsilon )-{V}_{t1}({\mu }_{0},{\sigma }_{0},{\varepsilon }_{0})$$

The hypothesis is that interrogating the ferromagnetic specimen with different excitation frequencies could result in changes in the magnetic permeability of the specimen. It is anticipated that the change in relative complex permeability change |*δμ*| could be reconstructed from the amplitude of the induced voltages, if the electrical conductivity is insensitive within the range of interrogating frequencies. Hence a frequency difference technique is applied to reveal the change in frequency-dependent permeability. This technique is applied in the following manner:

5 $${\rm{\Delta }}{V}_{f1,f2}(\delta \mu ,\delta \sigma ,\delta \varepsilon )={V}_{f2}(\mu ,\sigma ,\varepsilon )-\frac{{V}_{f2}({\mu }_{0},{\sigma }_{0},{\varepsilon }_{0})}{{V}_{f1}({\mu }_{0},{\sigma }_{0},{\varepsilon }_{0})}\cdot {V}_{f1}(\mu ,\sigma ,\varepsilon )$$

where *V*_*f*1_ and *V*_*f*2_ are the induced voltages at a reference frequency *f*_1_ and an interrogating frequency *f*_2_. The frequency difference measurement is Δ*V*_*f*1,*f*2_. The reference frequency *f*_1_ is 5 kHz. The changing frequency *f*_2_ varies from 1 to 100 kHz. In this study, the reference frequency is chosen empirically, considering that the measurement system operates within the range of 1 kHz to 100 kHz with a uniform drive current of 100 mA. It is recommended practice to avoid operating the system at its limits. Several frequencies including 5 kHz, 10 kHz, 15 kHz, 20 kHz and 30 kHz have been investigated as suitable reference frequencies based on the amplitude of signals from the receiving coils. With a constant current source, utilising high frequency excitation increases the amplitude and stability of the signals from receiving coils. It is concluded that 5 kHz provides not only uncompromising signals and also greater depth penetration in comparison with other frequencies, thus allowing a low frequency component of frequency difference imaging, should the internal structure of a ferromagnetic material be of interest.

Once the reference frequency is chosen, a frequency difference measurement is applied because systematic errors and drifts can be better suppressed^14^. A scaling factor is necessary to calibrate the measurements in the same range^15^. In this case, the scaling factor is taken from the ratio of the induced voltages $(\frac{{V}_{f2}({\mu }_{0},{\sigma }_{0},{\varepsilon }_{0})}{{V}_{f1}({\mu }_{0},{\sigma }_{0},{\varepsilon }_{0})})$ when the system is excited at a reference frequency *f*_1_ and a sweep of frequency *f*_2_ in a free space.

The inverse problem is solved and all the images included in this paper reconstructed using a Total Variation reconstruction algorithm^16^.

## Results

**Figure 1:**
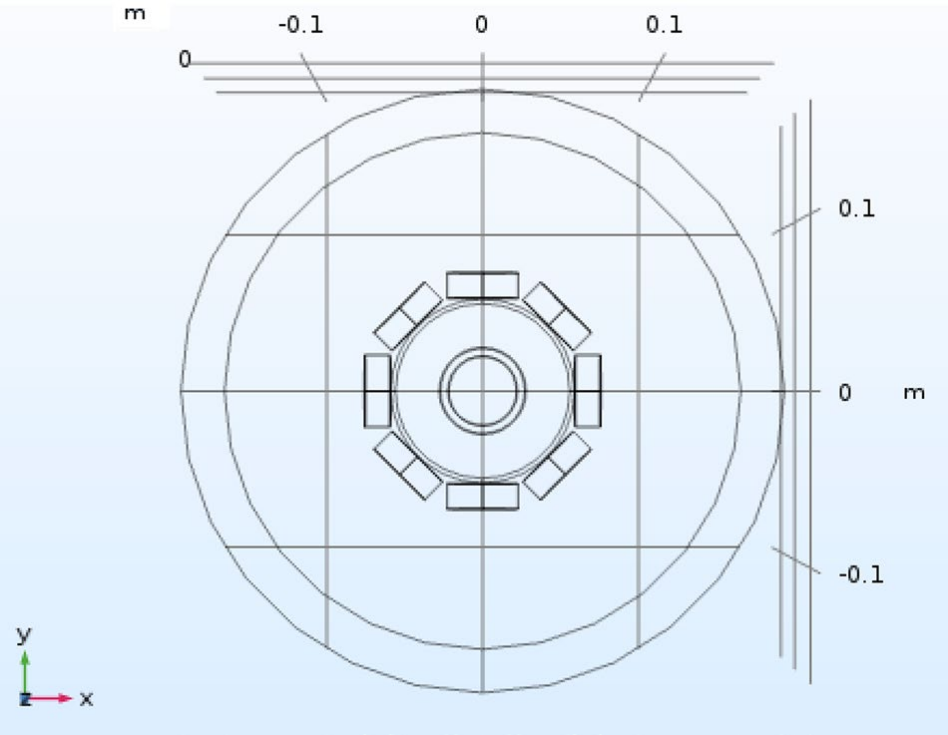
Top view of a tube (OD 50.76 mm, height of 135.00 mm) placed in the centre of an eight-coil MIT imaging region.

**Figure 2:**
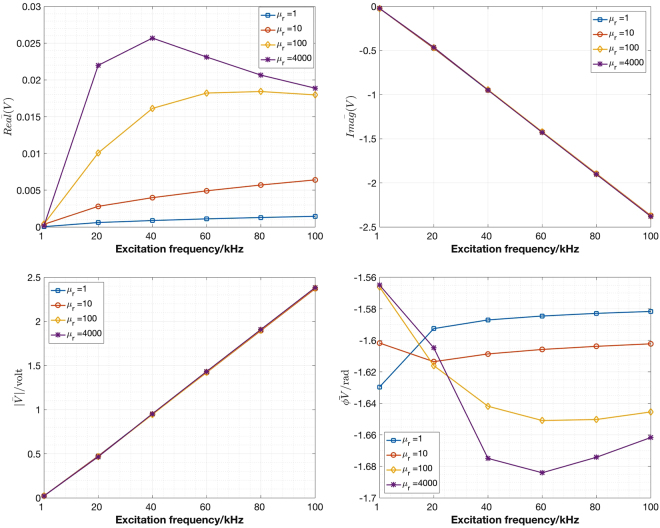
Mean values of the real part (**A**), imaginary part (**B**), amplitudes (**C**) and phase angles (**D**, in radian) of induced complex voltages at different single excitation frequencies, where the phantom tubes have a conductivity of 1.3 × 10^6^ *S*/*m* and different relative permeability, of 1, 10 100 and 4000 respectively.

Figure 1 shows a top view of the simulation setup, where a tube with an outer diameter (OD) of 50.76 mm and a height of 135.00 mm is placed in the centre of a MIT imaging region surrounded by eight inductive coils. The entire model is simulated in a free space. The conductivity of the tube is 1.3 × 10^6^ *S*/*m*. The relative permeability of the tubes are, respectively, 1, 10, 100, 4000 in each simulation. In cases whereby the relative permeability is equal to 1, the tube is of conductive and non-magnetic nature. The choice of these relative permeability values arises from the fact that carbon steel and electrical steel have a relative permeability close to 100 and 4000, respectively. The following excitation frequencies (1, 20, 40, 60, 80 and 100 kHz) are provided to drive the system for each relative permeability scenario. These frequencies are also within the capability of the measurement system. The purpose of this simulation is to investigate the effect of differential permeability on the induced voltages under the same excitation frequency. For complex induced voltages, the mean values of the real part, imaginary part, amplitudes and phase angles of the voltages are shown in Fig. 2. For future work, it would be desirable to carry out frequency spectroscope simulations so that the permeability change due to frequency different can be quantitatively and qualitative characterised with known material permeability, thus offering a theoretical basis for the choice of reference frequency in a greater range.

In the first set of experiments, two cylindrical specimens are investigated: a non-magnetic (aluminium) tube and a ferromagnetic (electrical steel) tube. Both are conductive, whereas the aluminium tube has a relative permeability of 1 and the electrical steel has a relative permeability in the region of 4000. Two tubes are machined with the same dimensions, an outer diameter (OD) of 50.76 mm and a height of 70.00 mm.

**Figure 3:**
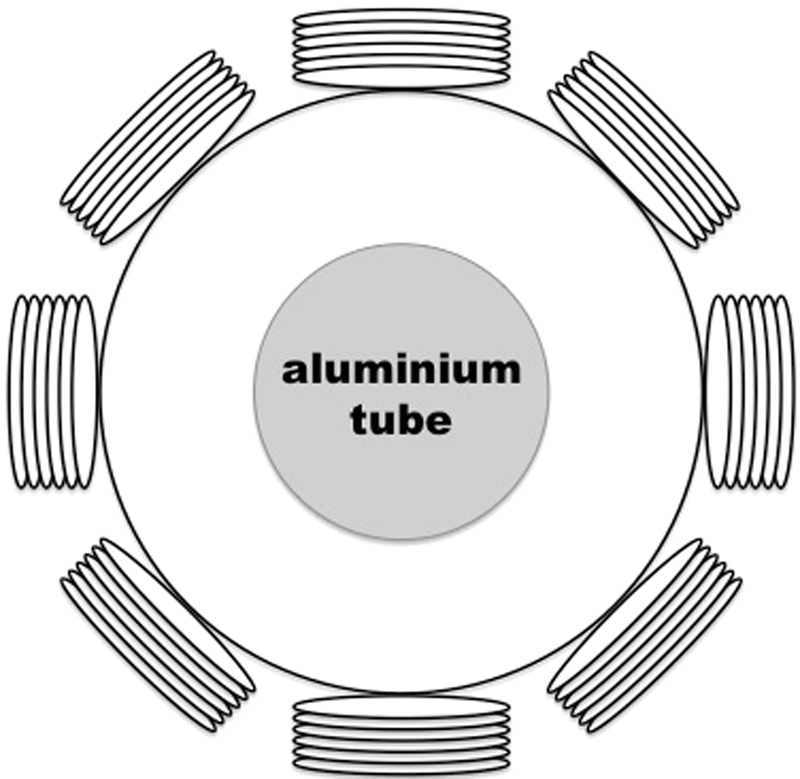
Experimental setup of an aluminium tube (OD50.76 mm) positioned in the centre of the imaging region.

**Figure 4:**
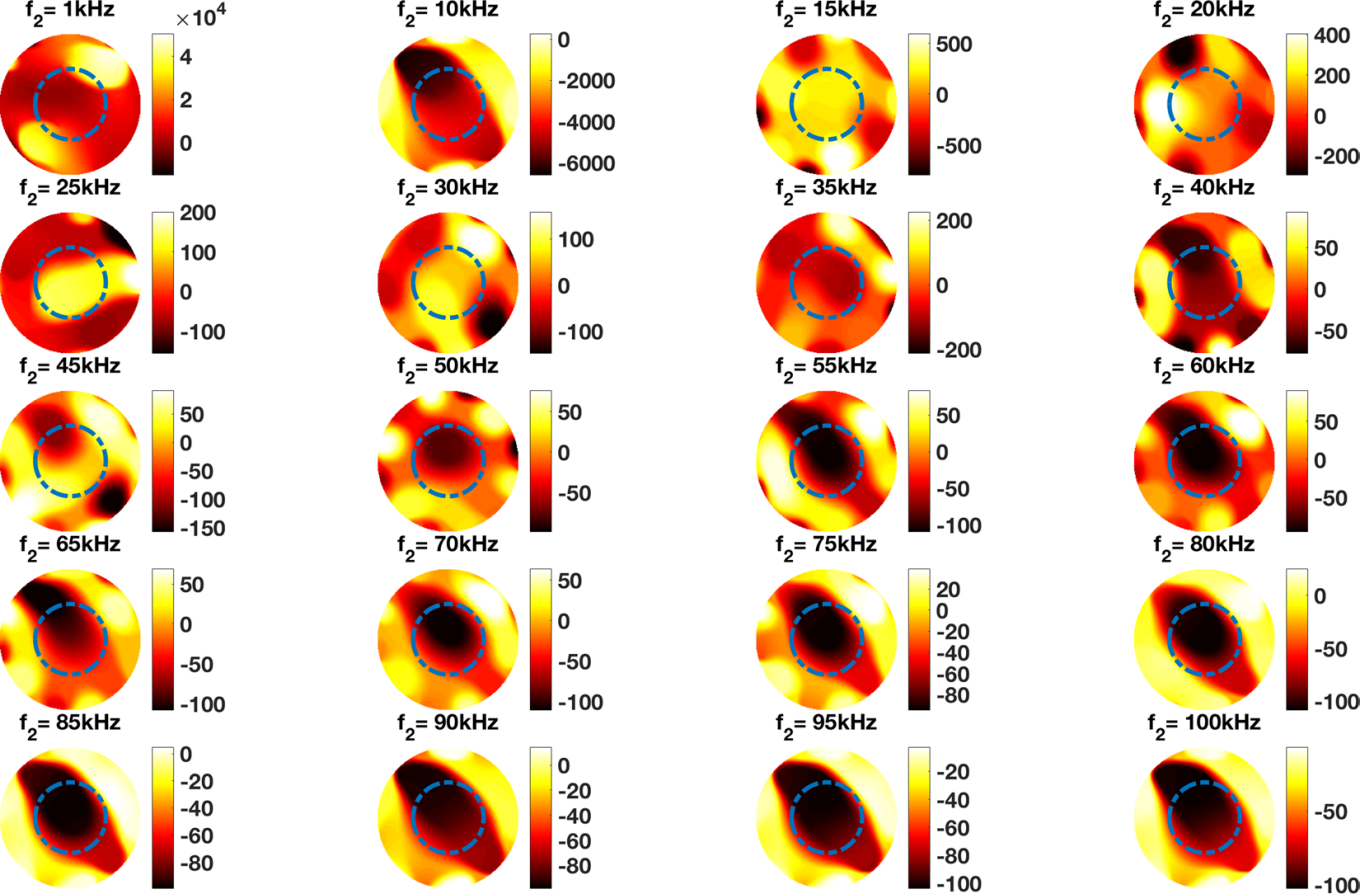
Reconstructed frequency difference images of an aluminium tube (OD50.76 mm) positioned in the centre of the imaging region (experimental setup shown in Fig. 3).

Figure 3 shows the experimental setup of an aluminium tube positioned in the centre of the imaging region and Fig. 4 shows the frequency difference imaging results, with the dashed circles indicating the location of the tube (calculated based on the dimensions of the tube) with respect to the dimensions of the imaging region. A series of experiments are conducted using non-magnetic metallic samples such as aluminum and copper using frequency difference imaging. As with the results shown in Fig. 4, aside from minor differences due to skin depth, no meaningful image could be reconstructed with other non-magnetic metals. In subsequent experiments, ferromagnetic samples are used such that their frequency-dependent permeability can enable frequency difference imaging. Colour bars representing the amplitudes of relative reconstructed permeability values are shown, where applicable, throughout the remainder of this paper.

**Figure 5:**
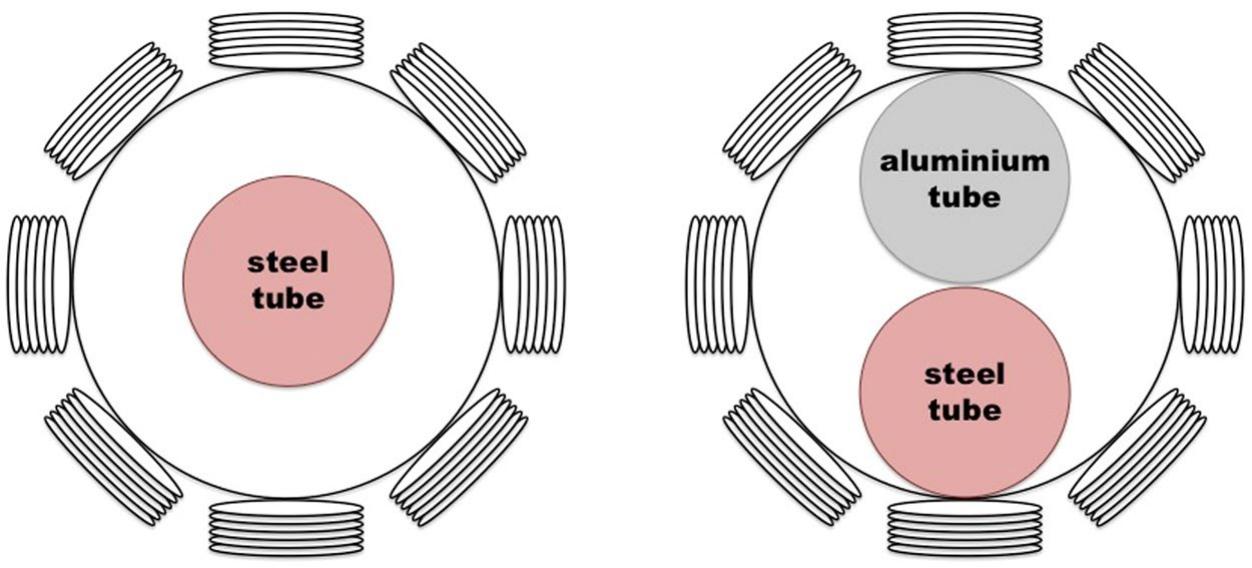
Experimental setup of a steel tube (OD50.76 mm) positioned in the centre of the imaging region (**A**), and an aluminium tube (OD50.76 mm) and a steel tube (D50.76 mm) positioned side by side in the imaging region (**B**).

**Figure 6:**
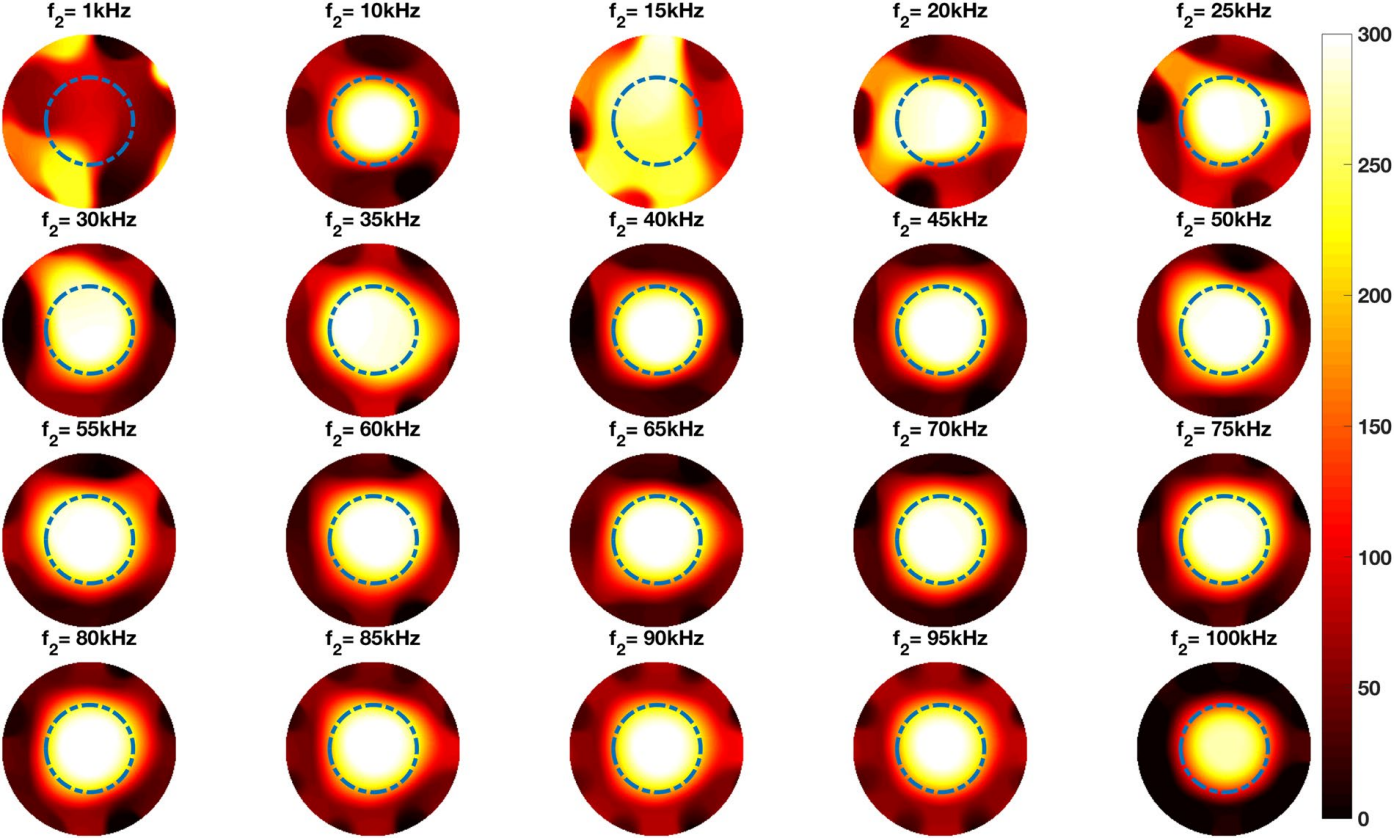
Reconstructed frequency difference images of a steel tube (OD50.76 mm) positioned in the centre of the imaging region (experimental setup shown in Fig. 5A).

**Figure 7:**
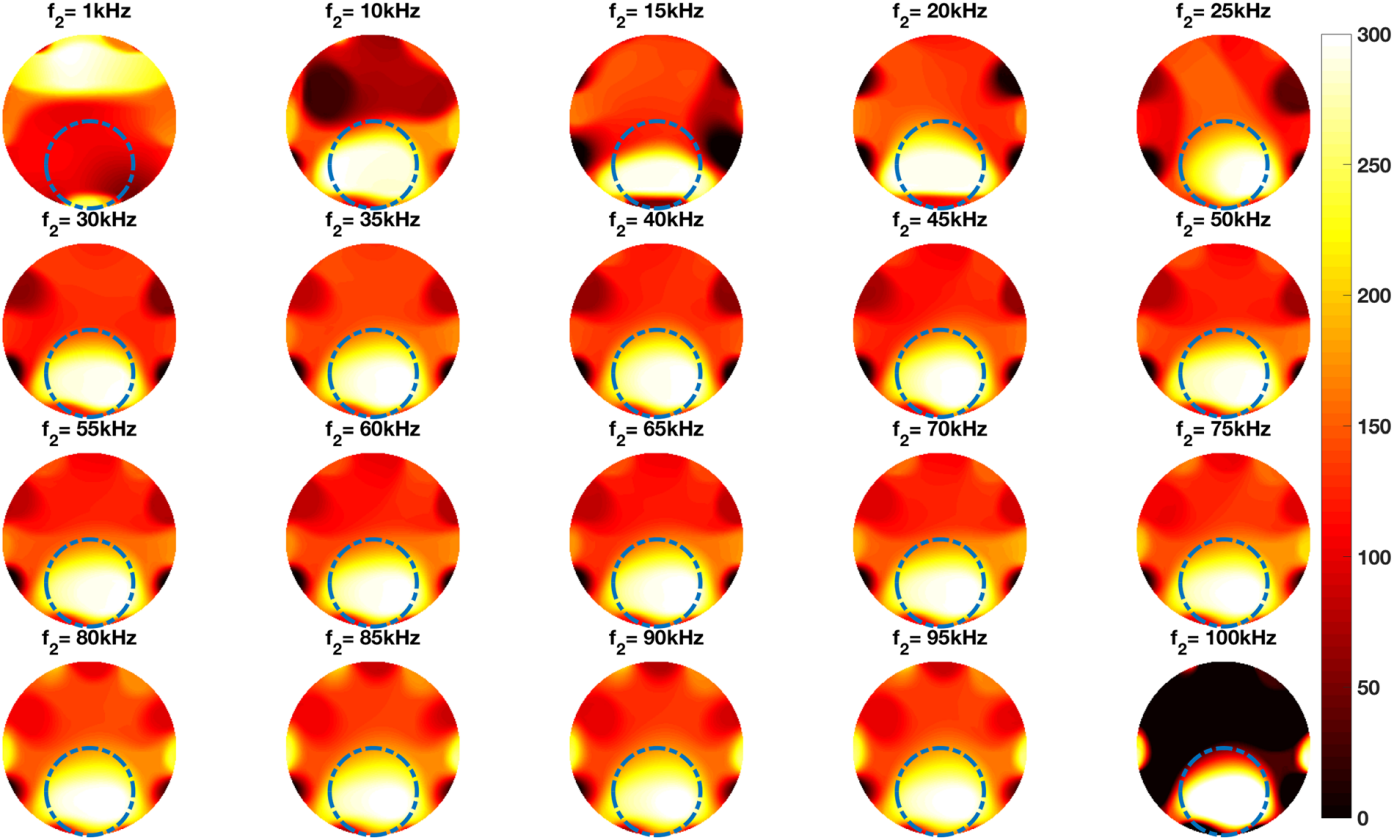
Reconstructed frequency difference images of an aluminium tube (OD50.76 mm) and a steel tube (OD50.76 mm) positioned side by side in the imaging region (experimental setup shown in Fig. 5B).

Figure 5A shows the experimental setup of a steel tube positioned in the centre of the imaging region. Figure 5B shows the same steel tube and an aluminium tube positioned side by side in the imaging region. The reconstructed frequency difference images for both cases are shown in Figs 6 and 7, respectively.

In the second set of experiments, specimens with different geometries are investigated: a non-magnetic (aluminium) rod and a ferromagnetic (electrical steel) cube. Both are similarly conductive, although the aluminium tube has a relative permeability of 1 and the electrical steel has a relative permeability in the region of 4000. The aluminium rod has a diameter of 42.00 mm and a height of 44.00 mm. The steel cube has dimensions of 38.00 × 38.00 × 38.00 mm.

**Figure 8:**
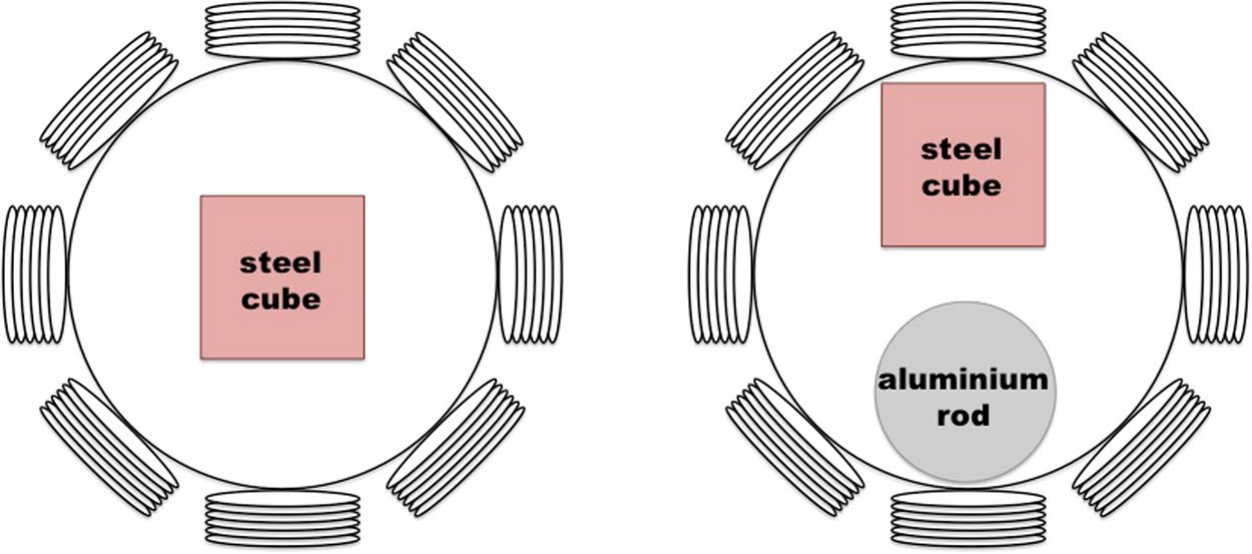
Experimental setup of a steel cube (side length 38.00 mm) positioned in the centre of the imaging region (**A**), and an aluminium tube (D42.00 mm, H44.00 mm) and the same steel tube positioned side by side in the imaging region (**B**).

**Figure 9:**
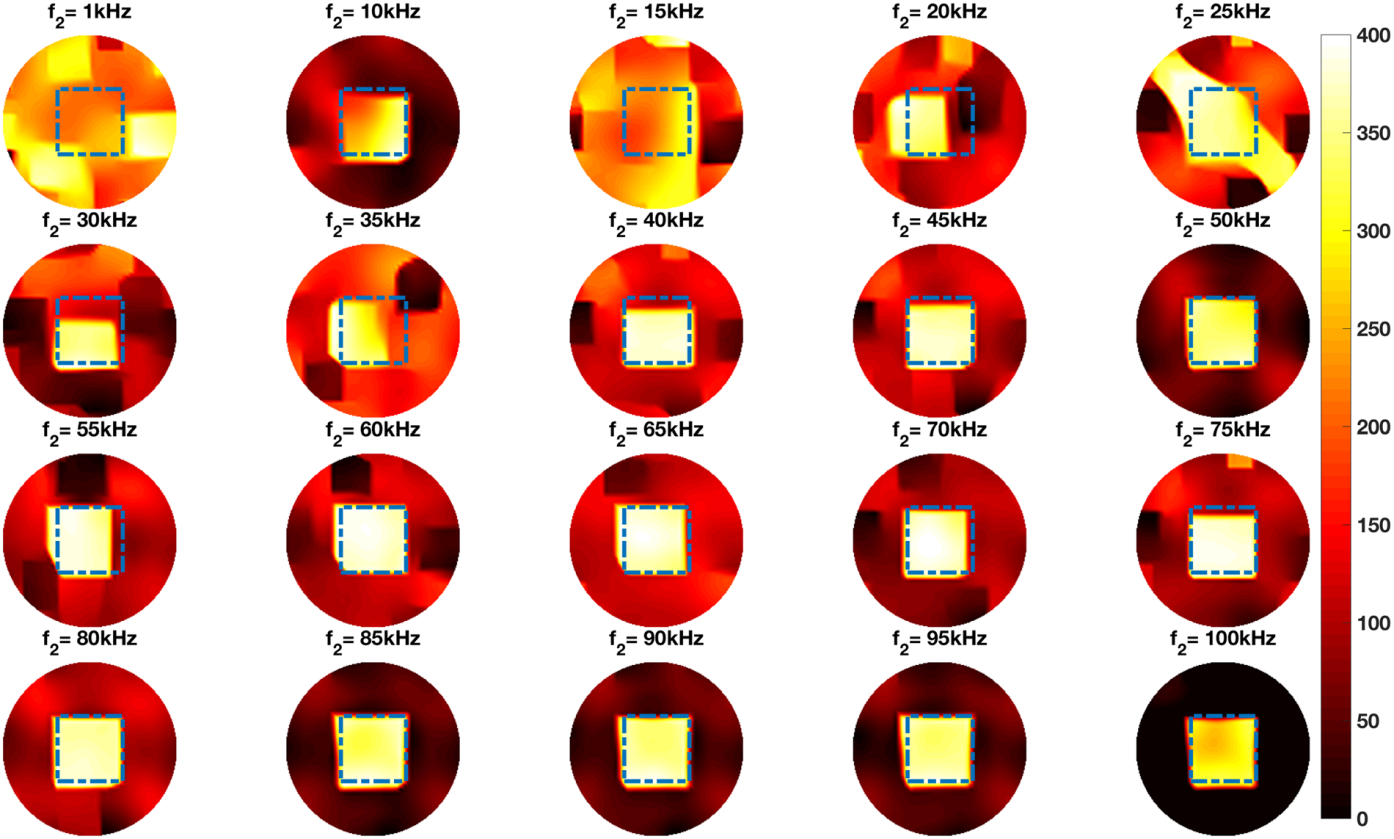
Reconstructed frequency difference images of a steel cube (side length 38.00 mm) positioned in the centre of the imaging region (experimental setup shown in Fig. 8A).

**Figure 10:**
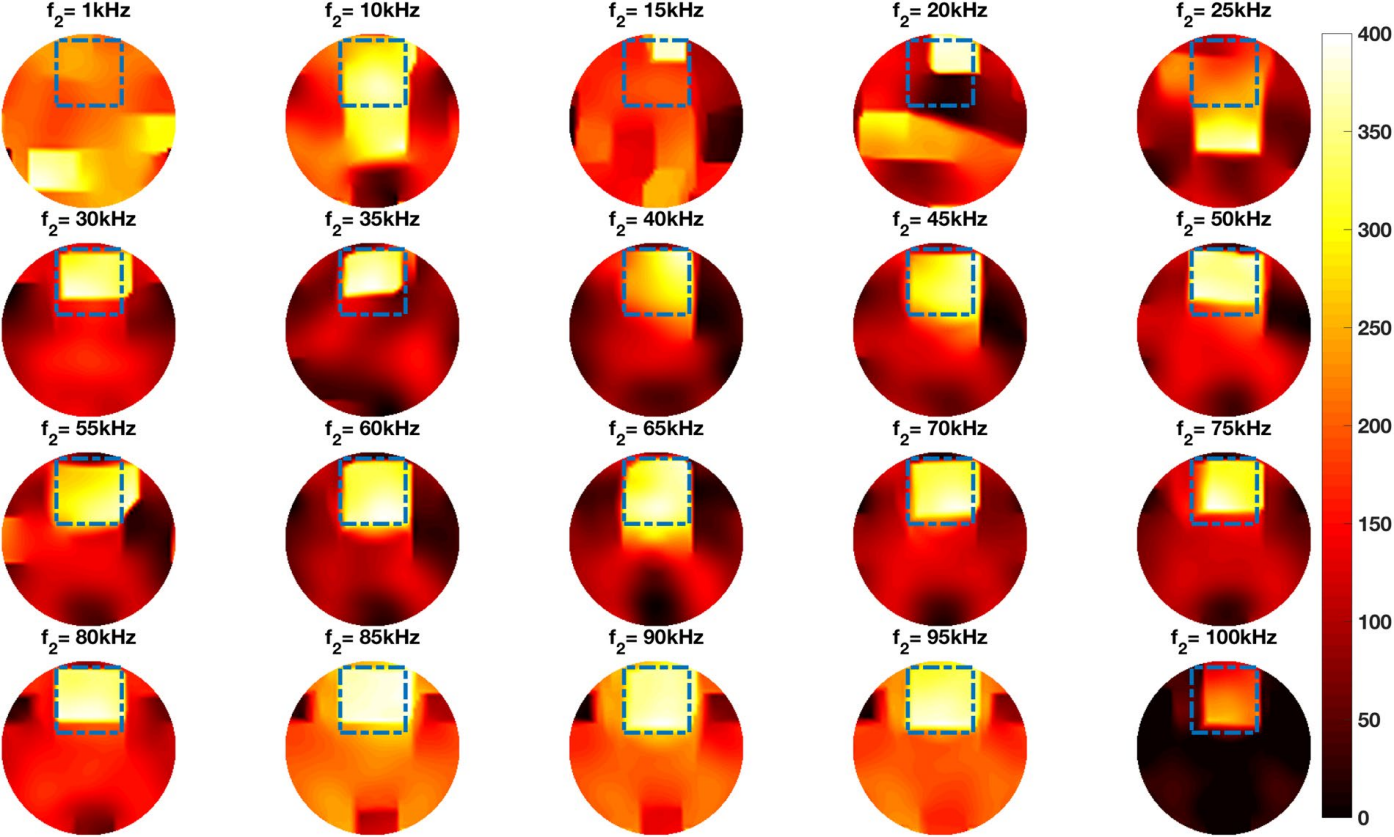
Reconstructed frequency difference images of an aluminium rod (D42.00 mm, H44.00 mm) and a steel cube (side length 38.00 mm) positioned side by side in the imaging region (experimental setup shown in Fig. 8B).

Figure 8A shows the experimental setup of the steel cube positioned in the centre of the imaging region. Figure 8B shows the same cube and the aluminium rod positioned side by side in the imaging region. The reconstructed frequency difference images for these setups are shown in Figs 9 and 10, respectively. In each figure, the dashed squares and circles indicate the location of the rod and cube (calculated based on the true dimensions of the rod and cube) with respect to the dimensions of the imaging region. Frequency difference imaging clearly illustrates the magnetic permeability of the specimen and its variation with frequency.

## Discussion

The experimental configurations and comparisons can be categorised as follows. Firstly, a single cylindrical specimen with different electromagnetic properties – non-magnetic and ferromagnetic – positioned in the centre of the imaging region (Figs 4 and 6). It can be seen from Figs 4 and 6 that an aluminium tube cannot be reconstructed whereas a steel tube can be reconstructed using frequency difference MIT measurements. A more consistent image quality is observed when the interrogating frequency is above 30 kHz in Fig. 6. The purpose of this experiment is to show that for the same imaging specimen geometry located in the same position, the frequency-dependent character is observed in a ferromagnetic specimen using spectroscopy imaging. This is observed both for a ferromagnetic tube (Figs 6 and 7) and a ferromagnetic cube (Figs 9 and 10) positioned in two different positions (centre and off-centre) of the imaging region. The imaging results for these tests demonstrate that the frequency-dependent permeability is independent of the specimen position and geometry. Thirdly, reconstructed images in Figs 6 and 9 suggest that similar image qualities are obtained when changing the shape of the specimen from cylindrical to cubical. When comparing the images in Figs 6, 7, 9 and 10, it is evident that when the specimen is shifted off-centre, images can still be reconstructed reflecting the corresponding positions of the imaging specimen. Finally, the effect of a non-magnetic specimen in the presence of a ferromagnetic specimen in the imaging region is shown (Figs 7 and 10). These results show that only the ferromagnetic specimen is reconstructed. This further supports the hypothesis that within the frequency of 1–100 kHz, the permeability is frequency-dependent whereas the conductivity is insensitive.

In summary, this study presents a magnetic induction spectroscopy for the imaging of ferromagnetic materials. With differential frequency measurements, the need to collect reference data in a traditional time difference domain is eliminated. This approach is therefore superior for online structural integrity monitoring and characterisation of ferromagnetic materials.
